# The Time-Feature of Uric Acid Excretion in Hyperuricemia Mice Induced by Potassium Oxonate and Adenine

**DOI:** 10.3390/ijms21155178

**Published:** 2020-07-22

**Authors:** Shaoshi Wen, Dan Wang, Haiyang Yu, Mengyang Liu, Qian Chen, Ruixia Bao, Lin Liu, Yi Zhang, Tao Wang

**Affiliations:** 1 Tianjin State Key Laboratory of Modern Chinese Medicine, Tianjin University of Traditional Chinese Medicine, 312 Anshanxi Road, Nankai District, Tianjin 300193, China; 13821190632@126.com (S.W.); liumengyang0212@126.com (M.L.); lemonadelinliu@163.com (L.L.); 2 Key Laboratory of Pharmacology of Traditional Chinese Medical Formulae (Tianjin University of Traditional Chinese Medicine), Ministry of Education, 312 Anshanxi Road, Nankai District, Tianjin 300193, China; nkwangdan@163.com (D.W.); hyyu@tjutcm.edu.cn (H.Y.); 3 Institute of Traditional Chinese Medicine, Tianjin University of Traditional Chinese Medicine, 312 Anshanxi Road, Nankai District, Tianjin 300193, China; serafinachen@163.com (Q.C.); bz93171125@163.com (R.B.)

**Keywords:** hyperuricemia, mouse model, uric acid excretion, renal injury, urate transporter

## Abstract

Hyperuricemia is an important risk factor of chronic kidney disease, metabolic syndrome and cardiovascular disease. We aimed to assess the time-feature relationship of hyperuricemia mouse model on uric acid excretion and renal function. A hyperuricemia mouse model was established by potassium oxonate (PO) and adenine for 21 days. Ultra Performance Liquid Chromatography was used to determine plasma uric acid level. Hematoxylin-eosin staining was applied to observe kidney pathological changes, and Western blot was used to detect renal urate transporters’ expression. In hyperuricemia mice, plasma uric acid level increased significantly from the 3rd day, and tended to be stable from the 7th day, and the clearance rate of uric acid decreased greatly from the 3rd day. Further study found that the renal organ of hyperuricemia mice showed slight damage from the 3rd day, and significantly deteriorated renal function from the 10th day. In addition, the expression levels of GLUT9 and URAT1 were upregulated from the 3rd day, while ABCG2 and OAT1 were downregulated from the 3rd day, and NPT1 were downregulated from the 7th day in hyperuricemia mice kidney. This paper presents a method suitable for experimental hyperuricemia mouse model, and shows the time-feature of each index in a hyperuricemia mice model.

## 1. Introduction

With the improvement in people’s living standard and the change in diet structure, the incidence of hyperuricemia is increasing. A large number of clinical research results show that hyperuricemia is closely related to cardiovascular disease, immune system disorders, chronic renal failure [1,2]. Therefore, the basic research of anti-hyperuricemia is great significance to prevent the occurrence and development of these diseases.

Hyperuricemia can lead to uric acid deposition in renal tissue, leading to acute kidney injury (AKI). AKI exhibited renal tubule dilation, inflammatory cell infiltration, inconspicuous of boundaries between adjacent proximal tubule cells, and cell necrosis [3]. At present, AKI is diagnosed based on a dynamic increase in serum creatinine and decreased urine output. Serum cystatin C and serum or urine neutrophil gelatinase-associated lipocalin (NGAL) have been shown to predict or diagnose AKI [4]. Urine kidney injury molecule 1 (KIM-1), urine transferrin, albuminuria, circulating angiopoietin-2, L-FABP, vanin-1, and IgG are also important predictors [5,6,7,8,9,10,11].

The main cause of hyperuricemia is the increase in uric acid production, which may be caused by genetic factors or due to excessive purine precursor intake from the diet. Another major pathological change in hyperuricemia is uric acid excretion default [12]. To date, allopurinol and febuxostat have been used to inhibit the excessive production of UA and achieved an excellent therapeutic effect [13,14]. Kidney is the most important organ to excrete UA. Clinically, the most common causes of hyperuricemia are insufficient excretion of UA in the kidney, downregulation of UA secretion transporter (urate-anion transporter 1 (URAT1), glucose transporter 9 (GLUT9), et al.) [15,16] and up-regulation of reabsorption transporter (ATP-binding cassette super family G number 2 (ABCG2), organic anion transporter 1 (OAT1), sodium-dependent phosphate cotransporter 1 (NPT1), et al.) [17,18,19]. However, drugs with improving effects on UA excretion are rare.

Animal models play an important role in the basic research of UA regulatory substances. To date, zebrafish [20], birds (hawk and broiler) [12,21], pig [22], tree shrew [23], rat [24] and mouse [25] have been used as experimental animals. Among them, due to practical and economic reasons and species differences with humans, mice and rats are more widely used.

According to the literature, potassium oxonate (PO) (an uricase inhibitor) with or without high purine food is usually used as hyperuricemia inducer in rats/mice, but the experimental protocols are different. For example, the duration of modeling was 7 [26] or 14 days [27], PO was orally [28] or intraperitoneally injected [26], PO dose was 200 [29], 250 [30] or 300 mg/kg [31], etc. The administration time is not uniform, resulting in a large difference between the data by different research groups, with some opposite results. For example, Chen et al. established the hyperuricemia mouse model by PO for 14 days, and the expression of ABCG2 was upregulated in the model group [27], however, Kodithuwakku et al. established the hyperuricemia mouse model by PO for 7 days, and the expression of ABCG2 was downregulated in the model group [32].

To clarify the time-feature of the hyperuricemia mouse model, we continuously administrated mice with PO and adenine for 21 days. The bodyweight, amounts of food and water intake, plasma uric acid level, expression of uric acid transporter and renal histopathological changes were detected in various time intervals. This paper suggested a suitable approach for experimental hyperuricemia mouse model.

## 2. Results

### 2.1. The General Condition of Hyperuricemia Mice Induced by Potassium Oxonate (PO) and Adenine

During the model experiment, the mice in the normal group had a good mental state, active behavior and smooth and shiny fur. However, most of the mice in the model group showed a poor mental state, became emaciated, listless and addicted to sleeping, and hair color withered.

### 2.2. The Time-Feature on Bodyweight, Water Intake and Food Intake in Hyperuricemia Mice Induced by PO and Adenine

**Figure 1:**
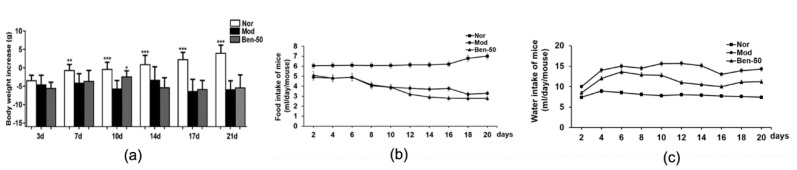
Bodyweight, water intake and food intake changing in hyperuricemia mice. (**a**) Bodyweight; (**b**) Food intake; (**c**) Water intake; Nor, Normal control group; Mod, PO-induced hyperuricemia group; Ben-50, positive control benzbromarone group. Values represent the mean ± SD of determinations (*n* = 12); * *p* < 0.05, ** *p* < 0.01, *** *p* < 0.001 vs. hyperuricemia group.

As shown in Figure 1, the bodyweight of mice was recorded; the bodyweight in the normal group increased gradually, while the bodyweight in the model group decreased obviously. Compared with the model group, the mice gained bodyweight within 10 days, decreased on the 10th day, and no significant change was found after 14 days administration of benzbromalone (50 mg/kg *p.o.*). The mice in the model group drank more water compared with the normal group, and the water intake of benzbromalone mice decreased compared with the model group. In addition, compared with the normal group, the food intake of the model group was significantly lower, and there was no significant change in the food intake of benabromalone mice compared with the model group.

### 2.3. The Time-Feature on the Level of Uric Acid in Plasma of Hyperuricemia mMce Induced by PO and Adenine

**Figure 2:**
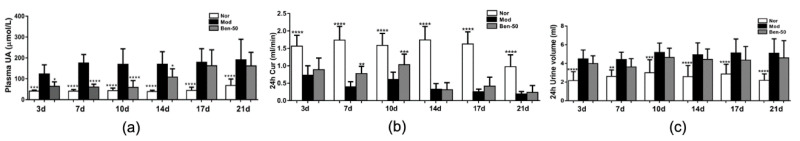
Plasma urate levels and excretion of urate in PO- and adenine-induced hyperuricemia mice. (**a**) Plasma uric acid levels after administration; (**b**) 24 h Cur; (**c**) Urine volume. Nor, normal control group; Mod, PO-induced hyperuricemia group; Ben-50, positive control benzbromarone group. Values represent the mean ± SD of determinations (*n* = 12); * *p* < 0.05, ** *p* < 0.01, *** *p* < 0.001, **** *p* < 0.0001 vs. hyperuricemia group.

During the model experiment, compared with the normal group, the uric acid level in the model group was significantly higher. As shown in Figure 2 and Table 1, on 7th day, the level of uric acid in the model group plasma was about three times that in the normal group. Compared with the model group, the level of uric acid in the plasma of the mice decreased significantly in the benabromalone group.

In addition, compared with the normal group, the urine volume of the model group increased significantly (Figure 2). To evaluate uric acid excretion in hyperuricemia mice. Cur was calculated. In the hyperuricemia group, 24 h Cur was significantly decreased compared with normal mice, while Cur were partially recovered in hyperuricemia mice treated with benzbromarone (Figure 2).

### 2.4. The Time-Feature on Renal Morphology of Hyperuricemia Mice Induced by PO and Adenine

**Figure 3:**
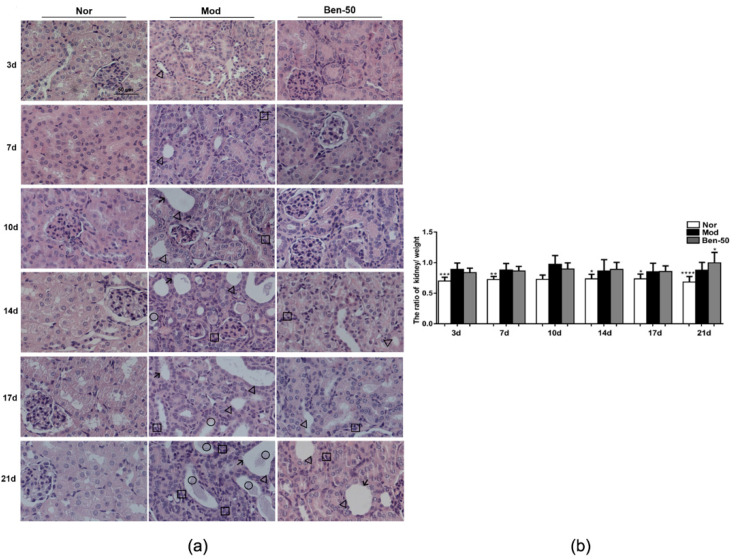
Kidney histological alterations in adenine- and PO-induced hyperuricemia mice. (**a**) Kidney sections for H&E staining (400×). Nor, normal control group; Mod, adenine and PO induced hyperuricemia group; Ben-50, positive control benzbromarone group. Black arrow, necrotic tubular epithelial, Black triangle, tubular ectasia, Black circle, corpora amylacea, Black square, inflammatory cell infiltration; (**b**) Kidney to body ratio of hyperuricemia mice induced by PO and adenine, Nor, normal control group; Mod, adenine and PO induced hyperuricemia group; Ben-50, positive control benzbromarone group. Values represent the mean ± SD of determinations (*n* = 12); * *p* < 0.05, ** *p* < 0.01, *** *p* < 0.001, **** *p* < 0.0001 vs. hyperuricemia group.

In model group, PO- and adenine-induced hyperuricemia mice exhibited severe kidney injury, and pathological change included renal tubule dilation, inflammatory cell infiltration, inconspicuous boundaries between adjacent proximal tubule cells, and cell necrosis (Figure 3). These phenomena became more and more serious with time. Benzbromarone treatment alleviated the hyperuricemia-induced renal damage. In addition, compared with the normal group, the kidney body ratio of the model group was upregulated (Figure 3).

### 2.5. The Time-Feature on the Expression of Urate Transporters Protein in Hyperuricemia Mice Induced by PO and Adenine

**Figure 4:**
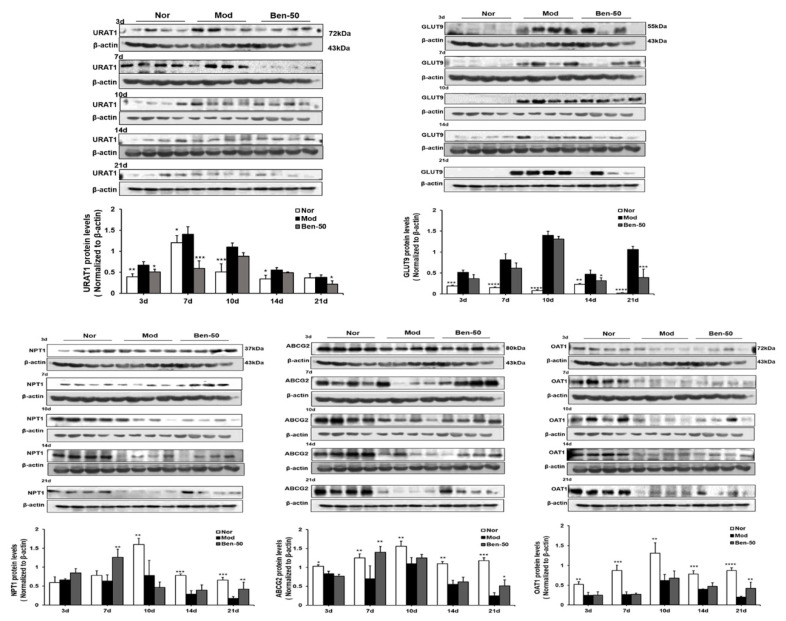
Renal urate transporters URAT1, GLUT9, OAT1, ABCG2, and NPT1 relative protein expression levels in PO- and adenine-induced hyperuricemia mice. Nor, normal control group; Mod, adenine- and PO-induced hyperuricemia group; Ben-50, positive control benzbromarone group. Values represent the mean ± SD of determinations (*n* = 12); * *p* < 0.05, ** *p* < 0.01, *** *p* < 0.001, **** *p* < 0.0001 vs. hyperuricemia group.

Renal URAT1, GLUT9, ABCG2, OAT1, and NPT1 protein expression were detected (Figure 4). Compared with the normal group, the expression level of URAT1 and GLUT9 protein in the kidney in the model group was significantly increased, especially on the 10th day (Table 2). In addition, the expression level of OAT1 protein in the kidney of the model group was significantly down-regulated. And the expression level of NPT1 did not change significantly within 7 days in the model group, but decreased greatly from the 10th day. Compared with the normal group, the expression level of ABCG2 was also significantly down-regulated in the model group.

### 2.6. The Time-Feature on Each Indicator in Hyperuricemia Mice Induced by PO and Adenine

**Figure 5:**
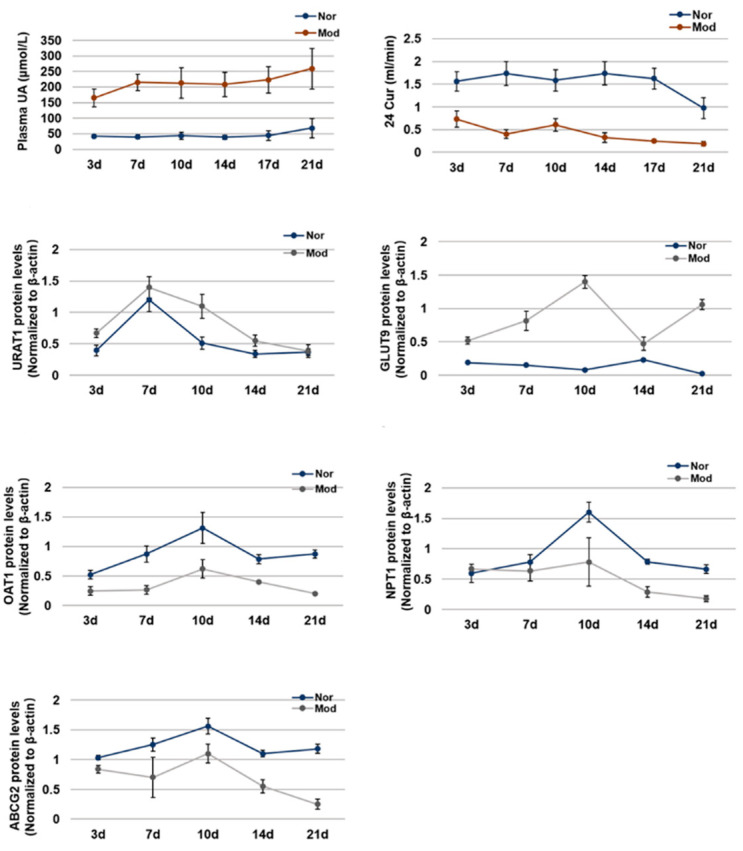
The time-feature of each index in PO- and adenine-induced hyperuricemia mice. Plasma uric acid levels, 24 h Cur changing curve; Expression of URAT1, GLUT9, OAT1, ABCG2, and NPT1 in mice kidney changing curve. Values represent the mean ± SD of determinations (*n* = 12). Nor, normal control group; Mod, adenine and PO induced hyperuricemia group.

We summarized the change trend with the time of plasma uric acid level, uric acid clearance rate, and the expression levels of ABCG2, URAT1, GLUT9, OAT1 and NPT1 in hyperuricemia mice (Figure 5). In hyperuricemia mice, the level of plasma uric acid was significantly higher and the clearance rate of uric acid was significantly lower than normal mice from the 3rd day to the 21st day. The expression of urate transporters GLUT9 and URAT1, which promote the reabsorption of uric acid, increased significantly from the 3rd day to 21st day in hyperuricemia mice. However, the expression of urate transporters ABCG2, OAT1 and NPT1, which promote the secretion of uric acid, decreased significantly from the 3rd day to the 21st day in hyperuricemia mice.

## 3. Discussion

The prevalence of hyperuricemia has increasing rapidly throughout the world. Recent research reported that a high uric acid level is the risk factor of gout, kidney stone, cardiovascular disease, and stroke [33]. Epidemiological studies have shown that the main causes of hyperuricemia are over-production and the low excretion of uric acid [34]. Kidney is the major organ which removes uric acid to maintain its balance in blood. Long-term high uric acid level leads to the impairment of renal filtration function and the decrease in the clearance rate of uric acid and creatinine.

A number of secretion and reabsorption transporter play important role in movement of uric acid from blood to urine. The secretion transporter includes OAT1 and organic anion transporter 3 (OAT3) localized on the basolateral membranes of proximal renal tubular epithelial cells, which transport urate from the interstitial space in the blood to proximal tubular epithelial cells, depending on the gradients for exchanged anions [20]. On the apical membrane of proximal tubular epithelial cells, ABCG2, NPT1, sodium-dependent phosphate transport protein 4 (NPT4) and multidrug-resistance-proteins MRP4 (ABCC4) have all been shown to contribute to the secretory transport of urate from proximal tubular epithelial cells into the tubule lumen [35]. The reabsorption transporter includes URAT1 localized on the apical membrane of renal proximal tubular epithelial cells, which transport uric acid from the tubule lumen to proximal tubule epithelial cells, the short isoform of GLUT9 on the apical membrane; its function is similar to URAT1 [16,25]. The long isoform of GLUT9 localized on the basolateral membranes of proximal tubular epithelial cells, which transport urate from renal tubular epithelial cells to the blood [2].

Mouse models of hyperuricemia can be divided into two main categories: mice with genetic modifications that result in hyperuricemia (genetically induced models) and mice that have been exposed to environmental factors that induce hyperuricemia (chemical inhibition of uricase-induced models and modification of the diet-induced models). The gene encoding uricase has not been inactivated in mice; genetic modification of the mouse orthologue *Uox* was an obvious target to establish a “human-like” model to study hyperuricemia. The average serum urate concentration of the *Uox^−/−^* mice was obviously higher than that in the wild-type controls [36]. The *Uox^−/−^* mice were viable and fertile but had a high mortality of 65% at 4 weeks of age because of severe nephropathy [36] and ~40% 5 weeks of age because of renal dysfunction, metabolic disorders associated with compromised insulin secretion, hypertension [37]. The chemical inhibition of uricase and modification of the diet is also widely used to induce increased serum urate concentrations in mice. PO, a selectively competitive uricase inhibitor, blocks the effect of hepatic uricase. The serum uric acid concentration of the hyperuricemia mouse model that was established by PO was significantly higher than that of the normal mice. This method can maintain a high level of uric acid for more than 12 h, and also has obvious damage in the kidney, but the death rate of the hyperuricemia mice was lower.

To the best of our knowledge, hyperuricemia does not occur in rodents because of the uricase gene. As they are easily housed and maintained characters, rats and mice are the preliminary choice to mimic the clinic hyperuricemia. Oral administration of PO and adenine is commonly used to induce hyperuricemia, but the time-feather of kidney damage and expression of uric acid transporter are unclear.

In this study, we conducted an oral administration with PO and adenine to treat 8-week-old mice for 21 days, and the kidney damage and expression of uric acid transporter were tested every 3/4 days. After 3 days administration, the level of plasma uric acid in model group was significantly elevated than untreated group, and increased gradually to the 7th day (about three times than normal group). From 7th to 21st day, the plasma uric acid levels remained stable. Within 10 days, the pathological observation showed slight renal lesions, such as slight cell necrosis and cell infiltration. From the 10th to 21st day, serious lesions were observed in the kidney, such as cell necrosis and cell infiltration increasing significantly. We further tested the time-feature of uric acid transporter in kidney. The expression of urate transporter URAT1 GLUT9 related to the reabsorption of uric acid in hyperuricemia mice was upregulated from the 3rd day. Urate transporters related to the secretion of uric acid also changed significantly in hyperuricemia mice, the expression of ABCG2 and OAT1 was downregulated from the 3rd day, and NPT1 was downregulated from the 7th day. We also observed that these urate transporters changed the most significantly on the 10th day. In this study, we investigated the time-features of hyperuricemia mice which were induced by PO combined with adenine for 21 days, especially the effects on renal injury and expression of uric acid transporter.

## 4. Materials and Methods

### 4.1. Ethical Approval

Ethical approval was obtained from the Science and Technological Committee and the Animal Use and Care Committee of TJUTCM (No. 201610006, 6 October 2016). All experiments were conducted in accordance with the Laboratory Animal-Guideline for Ethical Review of Animal Welfare issued by the National Standard GB/T35892-2018 of the People’s Republic of China and complied with the principles and standards for reporting animal experiments in *The International Journal of Molecular Sciences*.

### 4.2. Mice

Male, seven-week-old Kunming strain mice (*n* = 12; Beijing Vital River Laboratory Animal Technology Co., Ltd., Beijing, China) were used in the present study. All mice were allowed ad libitum access to a standard diet and drink, and housed at 25 ± 2 °C, humidity 60 ± 5% with 12:12-h light-dark cycle. All mice were acclimated to their living environment for 7 days before the experiments.

### 4.3. Adenine and PO Induced Hyperuricemia Mice

Hyperuricemia mouse model was established by oral administration of PO and adenine (Sigma-Aldrich Co., St. Louis, MO, USA). Male Kunming mice were randomized into several groups (*n* = 12): normal control, hyperuricemia control, and benzbromarone control (50 mg/kg). Benzbromarone is a clinical drug to promote the excretion of uric acid. In this study, Benzbromarone as a positive drug: it can reduce the serum uric acid level by promoting the excretion of uric acid, and can significantly reduce the serum uric acid level of mice after oral administration of PO and adenine for 1 and 2 h. The normal group and the model group were given the same amount of 5% acacia solution, and the benzbromarone group was given benzbromarone suspending in 5% acacia. After 1 h, except the normal group, the other groups were given the modeling drug (PO 200 mg/kg + adenine 50 mg/kg) for 21 days. All mice were anaesthetized using isoflurane (4–5% induction, 1.5–2% maintenance).

We used the orbital venous sinus approach to collect blood from mice. We set the different animal batches at different time nodes, as follow: the 3rd/7th/10th/14th/17th/21st day. Blood samples were collected 1 h after final administration for biochemical assays. When blood was collected, isoflurane was used for general anesthesia in mice. The 3rd/7th/10th/14th/17th/21st day after blood collection, the mice were then killed by carbon dioxide euthanasia. The process of carbon dioxide euthanasia was gradually filling in carbon dioxide; the filling rate is 10%-30% of the chamber volume. The left kidney was fixed in formalin solution, and the right kidney was frozen in liquid nitrogen and stored at −80 °C. On the day before sampling, mice were put into a metabolism cage to collect urine samples for 24 h.

### 4.4. Ultra Performance Liquid Chromatography (UPLC) for Uric Acid Level

Blood samples were collected in 1.5 ml EP tube and centrifuged at 5000 g for 10 min. A further pretreatment method was established using the literature method [38]. The UPLC analysis was performed using a Waters ACQUITY UPLC system (Waters, Milford, MA, USA). According to the method reported in the literature [39], the uric acid clearance rate (cur) was calculated as follows

Cur = Uv × Uur/Sur;

Sur, plasma uric acid level; Uur, urinary uric acid level; Uv, urine volume.

### 4.5. Histopathology of Renal Tissues

The kidney was fixed in neutral paraformaldehyde solution (4%) for tissue fixation. The organs were cut into appropriate sections, washed, dehydrated and embedded to make tissue wax. All specimens were cut into five micron-thick paraffin sections, and were stained with hematoxylin and eosin (H&E). After that, they were dehydrated, sealed and finally photographed with Axio Imager 2 (Zeiss, Oberkochen, Germany).

### 4.6. Western Blot Analysis

The method of protein samples preparation was reported in literature [29]. SDS-polyacrylamide gel (SDS-PAGE) electrophoresis was used to isolate protein and then transferred to PVDF membrane (Merck Millipore, Bedford, MA, USA). The target band was cut according to molecular weight and incubated at 4 C for overnight individually with antibodies. The next day, the blots were washed with TBST three times and incubated with the horseradish peroxidase coupled second antibody for 1 h at room temperature. Subsequently, the blots were washed with TBST three times, and then developed by Enhanced Chemiluminescence (Millipore Co., Ltd., Bedford, MA, USA). Finally, the protein bands were visualized with ChemiDoc MP Imaging System (Bio-Rad, Hercules, CA, USA). The gray levels of the bands were quantified using Image J analysis software.

### 4.7. Statistical Analysis

The statistical analyses were conducted by using GraphPad Prism (v.7; GraphPad Software, San Diego, CA, USA). Data depicted in graphs represent the means ± SD. Intergroup comparison was made using two-way ANOVA followed by Tukey’s test and the Normal distribution of the various values analyzed was confirmed by ANOVA. *p* < 0.05 was considered to represent a statistically significant difference.

## 5. Conclusions

The central aim of this study is to clarify the time-feature of uric acid excretion, renal pathological state, plasma uric acid level and uric acid transporter expression in hyperuricemia mice models. In this study, we found that the plasma uric acid level of hyperuricemia mice increased significantly from the 3rd day, and remained stable from the 7th day. Furthermore, hyperuricemia mice exhibited slight kidney injury from the 3rd day, and significantly deteriorated renal function from the 10th day. In addition, the expression levels of urate transporters GLUT9 and URAT1 were upregulated from the 3rd day, while urate transporters ABCG2 and OAT1 were downregulated from the 3rd day, and NPT1 were down regulated from the 7th day in hyperuricemia mice kidney.

## Figures and Tables

**Table 1:** The time-feature of plasma uric acid level and 24 h Cur in PO and adenine-induced hyperuricemia mice.

Admistration Time	Plasma Uric Acid Level		24 h Cur	
	Normal Group	Model Group	Normal Group	Model Group
3 days	42.026 ± 4.60 ***	123.446 ± 28.672	1.563 ± 0.213	0.732 ± 0.179
7 days	40.944 ± 7.260 ****	175.703 ± 26.675	1.736 ± 0.263	0.398 ± 0.096
10 days	43.955 ± 11.622 ****	169.457 ± 49.331	1.584 ± 0.231	0.607 ± 0.142
14 days	38.785 ± 6.508 ****	170.181 ± 39.331	1.740 ± 0.258	0.329 ± 0.106
17 days	44.402 ± 15.295 ****	179.640 ± 42.672	1.625 ± 0.232	0.251 ± 0.033
21 days	68.142 ± 30.834 ****	191.005 ± 65.312	0.975 ± 0.226	0.185 ± 0.051

Values represent the mean ± SD of determinations (*n* = 12); *** *p* < 0.001, **** *p* < 0.0001 vs. model group.

**Table 2:** The time-feature of renal urate transport-associated protein expression levels in PO- and adenine-induced hyperuricemia mice.

Admistration Time	Renal URAT1 (WB)		Renal GLUT9 (WB)		Renal ABCG2 (WB)	
	Normal Group	Model Group	Normal Group	Model Group	Normal Group	Model Group
3 days	0.395 ± 0.071 **	0.672 ± 0.085	0.193 ± 0.017 ***	0.517 ± 0.055	1.034 ± 0.045 *	0.837 ± 0.065
7 days	1.214 ± 0.172 *	1.407 ± 0.184	0.152 ± 0.017 ****	0.818 ± 0.145	1.252 ± 0.110 **	0.713 ± 0.338
10 days	0.517 ± 0.191 ***	1.126 ± 0.096	0.083 ± 0.019 ****	1.407 ± 0.096	1.561 ± 0.131 **	1.108 ± 0.162
14 days	0.343 ± 0.896 *	0.552 ± 0.631	0.231 ± 0.019 **	0.472 ± 0.103	1.112 ± 0.055 **	0.552 ± 0.113
21 days	0.368 ± 0.120	0.382 ± 0.055	0.022 ± 0.012 ****	1.063 ± 0.075	1.179 ± 0.077 ***	0.254 ± 0.082

Values represent the mean of normalized to β-actin ± SD of determinations (*n* = 12); WB: Western blot; * *p* < 0.05, ** *p* < 0.01, *** *p* < 0.001, **** *p* < 0.0001 vs. model group.

## Abbreviations

PO	Potassium oxonate
UA	Uric acid
URAT1	Urate-anion transporter 1
GLUT9	Glucose transporter 9
ABCG2	ATP-binding cassette super family G2
NPT1	Sodium-dependent phosphate cotransporter 1
OAT1	Organic anion transporter 1
UPLC	Ultra Performance Liquid Chromatography
AKI	Acute kidney injury
NGAL	Neutrophil gelatinase associated lipocalin
KIM-1	Kidney injury molecule 1
